# Robust Direct Bandgap Characteristics of One- and Two-Dimensional ReS_2_

**DOI:** 10.1038/srep13783

**Published:** 2015-09-08

**Authors:** Zhi Gen Yu, Yongqing Cai, Yong-Wei Zhang

**Affiliations:** 1 Institute of High Performance Computing, Singapore 138632, Singapore

## Abstract

Two-dimensional (2D) transition-metal dichalcogenides (TMDs), most notably, MoS_2_ and WS_2_, have attracted significant attention due to their sizable and direct bandgap characteristics. Although several interesting MoS_2_ and WS_2_-based optoelectronic devices have been reported, their processability and reproducibility are limited since their electrical properties are strongly dependent of the number of layers, strain and sample sizes. It is highly desirable to have a robust direct bandgap TMD, which is insensitive to those factors. In this work, using density functional theory, we explore the effects of layer number, strain and ribbon width on the electronic properties of ReS_2_, a new member in the TMD family. The calculation results reveal that for monolayer ReS_2_, the nature (direct versus indirect) and magnitude of its bandgap are insensitive to strain. Importantly, the predicted bandgap and also charge carrier mobilities are nearly independent of the number of layers. In addition, the direct bandgap of ReS_2_ nanoribbons is only weakly dependent on their width. These robust characteristics strongly suggest that ReS_2_ has great potential for applications in optoelectronic nanodevices.

Recently, two-dimensional (2D) transition-metal dichalcogenides (TMDs), a new class of 2D materials with a chemical formula of MX_2_ (M = Mo, W, *et al.*, and X = S, Se, *et al.*), are considered as promising high-performance electronic and optoelectronic materials owing to their unique mechanical properties, chemical and environmental stability, and low threshold operating voltages^1^,^2^,^3^,^4^,^5^,^6^. MoS_2_ and WS_2_ are the two most widely studied TMDs. It is well-known that these TMDs exhibit a semiconducting characteristic with sizable and direct bandgap in their monolayer (ML) form. However, this direct bandgap characteristic changes to an indirect one in their multilayered or bulk form. Besides, with increasing the number of layer, there is a drastic reduction in bandgap. It is believed that such direct-to-indirect bandgap transition and drastic reduction in bandgap are caused by the quantum confinement along the thickness direction and the strong interlayer coupling, which can significantly change the electronic properties of these TMDs^7^. Also, when a small strain is applied to their monolayer form, a transition from a direct to an indirect bandgap also occurs^6^. In addition, studies also showed that the electronic properties of their one-dimensional form, that is, nanoribbons (NRs), are also sensitively dependent on the ribbon width^8^,^9^,^10^,^11^. Clearly, these changes in electronic properties with layer number, strain and sample dimensions pose great challenges in making robust electronic and optoelectronic devices based on those TMDs. Notably, only those ML TMDs with well-controlled strain and in-plane dimensions may be used for making optoelectronic devices to achieve desired optical bandgap characteristics and a high absorption and emission efficiency. Hence, in order to facilely and robustly fabricate optoelectronic devices using TMDs, it is important to find a novel TMD member whose electronic properties are insensitive to layer number, strain and NR width.

2D rhenium disulphide (ReS_2_), a new member in the TMD family, is a promising candidate as it exhibits a weak band renormalization, absence of interlayer registry and weak interlayer coupling arising from Peierls distortion of the 1T structure of ReS_2_^12^. Importantly, its monolayer form was recently experimentally produced through chemical exfoliation process and was found to retain the similar properties as the bulk^13^. In order to fully explore the potential of ReS_2_ in optoelectronic device applications, an in-depth understanding on the fundamental properties of ML, NR and multilayer ReS_2_ is indispensable. Similar research has been intensely focused on MoS_2_ and WS_2_ to explore their electrical properties for device applications^8^,^14^,^15^,^16^. In this study, we perform density functional theory (DFT) calculations to examine the effects of layer number, strain and NR width on the electronic properties of ReS_2_, with the aim of demonstrating their robustness against those factors.

## Results

### Lattice constants of bulk and ML ReS_2_.

**Figure 1:**
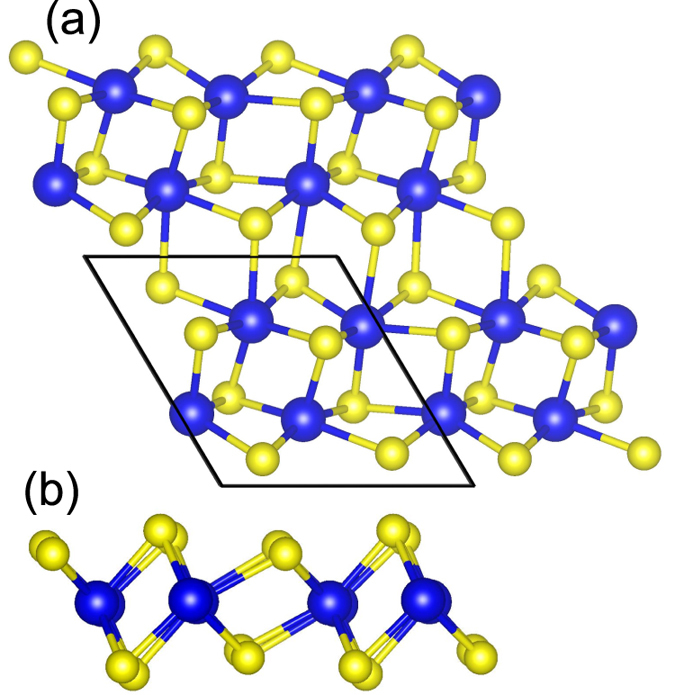
Top view (a) and side view (b) of the crystalline structures of distorted-1*T* (1*T*_*d*_) phases of ReS_2_ monolayer. Blue balls represent Re atoms and yellow balls represent S atoms.

**Figure 2:**
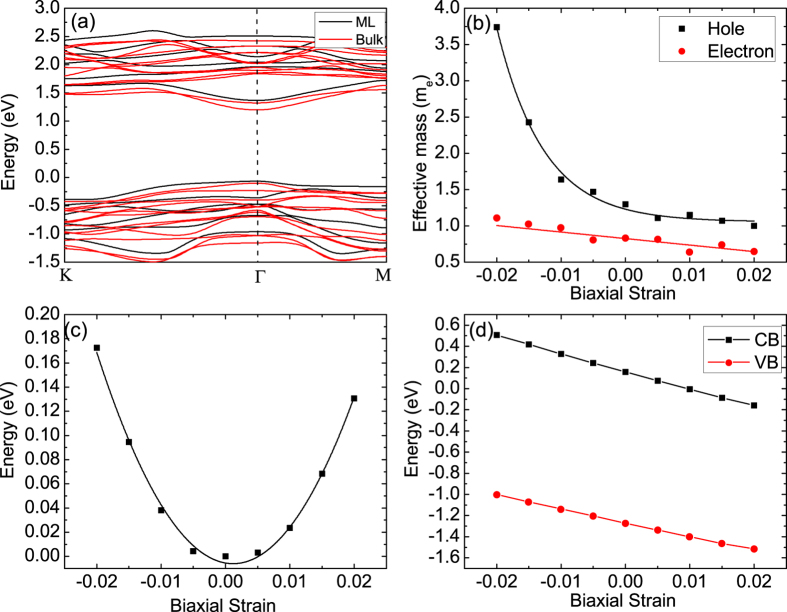
(**a**) Calculated band structures of ML (black) and bulk ReS_2_ (red). (**b**) Variation of electron (red) and hole (black) effective masses with biaxial strain in ML ReS_2_. (**c**) Variation of relative energy with biaxial strain in ML ReS_2_. Here, we set the relative energy of the free strain system is zero. (**d**) Band edge shift as a function of biaxial strain in ML ReS_2_.

The optimized ReS_2_ unitcell, which exhibits in a distorted octahedral layer structure with triclinic symmetry, is shown in Fig. 1. The calculated lattice constants are *a* = 6.51 Å, *b* = 6.41 Å, and *c* = 6.46 Å, respectively. These calculated lattice constants are in good agreement with experimental values (*a* = 6.45 Å, *b* = 6.39 Å, and *c* = 6.40)^17^. They are also exactly the same as the theoretical results reported by Tongay *et al.* (*a* = 6.51 Å, *b* = 6.41 Å)^12^. The S-Re bond length, Re-Re distance and S-S distance in one layer are 2.43/2.37 Å, 2.81 Å, and 2.88/3.25 Å, respectively. Note that the two values of S-Re bond length and S-S distance are due to the slight lattice distortion of S atoms in bulk ReS_2_. Hence, ReS_2_ has a unique crystal structure, which is distinctively different from other TMDs, in which their graphene-like hexagonal crystal structure is composed of layers of metal atoms sandwiched between layers of chalcogen atoms. From Fig. 2a, it is seen that bulk ReS_2_ is a direct gap semiconductor with a bandgap of 1.30 eV, which is close to the experimental value of 1.32 eV^18^. Based on the optimized lattice constants of bulk ReS_2_, we increase the interlayer spacing *c* to 20 Å to build a ML ReS_2_ model. It is found that the optimized lattice constants of ML ReS_2_ are *a* = 6.51 Å and *b* = 6.41 Å, which are exactly the same as the bulk. The same optimized lattice constants of bulk and ML ReS_2_ indicate that the interlayer coupling is negligible. This issue will be discussed in details later. The calculated band structure of ML ReS_2_ is shown in Fig. 2a. It is seen that ML ReS_2_ also shows a direct gap semiconductor characteristic with a bandgap of 1.43 eV, which is exactly the same as recently reported value^12^. It is worth noting that the nature of band structure of ReS_2_ is independent of the number of layer, which is in strong contrast to other TMDs, such as MoS_2_ and WS_2_, in which the nature of their band structures is strongly dependent on the number of layers^6^.

In order to confirm the weak interlayer coupling, we further calculate the formation enthalpies of bulk and ML ReS_2_, Δ*E*_*f*_, using following equation:


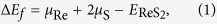


where 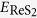 is the total energy of the bulk and ML ReS_2_, *μ*_Re,(S)_ denotes the corresponding atomic chemical potential calculated from metallic Re or solid S unitcell, respectively. In the calculation, we neglect the entropy contribution at 0 K. It should be noted that the calculated formation enthalpies of bulk and ML ReS_2_ are nearly identical (1.69 eV per ReS_2_ unit). Our DFT calculations without considering van der Waals (vdW) correction indeed confirm the weak interlayer coupling with the coupling energy of only 7 meV per unitcell, which is slightly smaller than the reported value of 18 meV^12^. It should be noted that the reported value of 18 meV was also calculated without vdW correction. We further calculate the interlayer coupling energy considering vdW correction by employing optB88-vdW functional, the calculated interlayer coupling energy is 0.265 eV. The difference of the interlayer coupling energy shows that vdW correction plays an important role in the theoretical calculation on multi-layer 2D materials^19^. In order to further verify the weak interlayer coupling, a more appropriate quantity to analyse is the surface energy for the exfoliation of the monolayer, *E*_*f*_ which is defined as 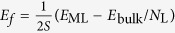, where *S* is the surface area of ML ReS_2_ unitcell and *E*_ML,bulk_ is the energy of ML and bulk ReS_2_ unitcell, respectively. *N*_L_ is the number of layers in each bulk ReS_2_ unitcell, in which, there are two layers in ReS_2_ unitcell. The calculated energy is 3.46 × 10^−4^ or 0.098 eV/Å^2^ without or with vdW correction, respectively. The low coupling energy and surface energy for the exfoliation of the monolayer indicate that single layer ReS_2_ can be easily exfoliated from the bulk^12^.

### Electronic structure and acoustic phonon-limited charge carrier mobility in ML ReS_2_

It is well-known that in inorganic semiconductor, the coherent wavelength of thermally activated electrons or holes at room temperature is much larger than lattice constants and is nearly equal to that of acoustic phonon modes in the center of first Brillouin zone (FBZ). Since the electron-acoustic phonon coupling dominates the scattering at the low energy regime^20^,^21^, the charge carrier mobility can be calculated by the deformation potential (DP), which has been extensively applied to calculate the mobility of 2D materials^22^. Based on the DP theory, the charge carrier mobility can be obtained by using the following formula^23^,^24^:


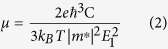


where m^*^ is the effective mass and *T* is the temperature, *E*_1_ is the DP constant which is defined as the shift of the band edges induced by strain, and *C* is the elastic modulus of uniformly deformed crystal under strain. For a 2D material, the in-plane stiffness *C* is defined as 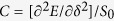, where *E* is the total energy of layered supercell, *δ* is the applied biaxial strain and *S*_0_ is the area of the optimized supercell. The effective mass m^*^ for charge transport is calculated by 
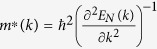
. All the above mentioned parameters can be obtained from our DFT calculations. In this study, only biaxial strains (including both compressive and tensile strains) are considered. The calculated electronic properties of ML ReS_2_ varying with strain are shown in Fig. 2b–d.

### Stability and edge energy of ReS_2_ NRs

ReS_2_ NRs can be directly produced by cutting ML ReS_2_ along x or y direction (see Supplementary Fig. S1 online), namely, RX-series or RY-series, respectively. First, we try to explore the stability of ReS_2_ NRs and figure out their energetically favorable configuration. It should be noted that no chemical functionalization, such as hydrogenation at the edges, is considered in this study although it was reported that hydrogenation at NR edges can make NRs more stable^11^.

For RY-series and RX-series, we select 5 different NR terminals and calculate their ground-state energies through optimizing structures by constraining the NR length in one unitcell. The distance between NR and its neighboring images is set to 18 Å and period boundary conditions (PBCs) are employed. Calculations on NRs with and without spin polarization are carried out to investigate the magnetic properties and the structure stability. According to the calculated density of states (DOS) (see Supplementary Fig. S2 online). It is seen that the calculated magnetic moments are zero for all selective NRs and the ground-state energies with and without spin polarization are identical. Accordingly, we only discuss the results without spin polarization in the following study. For comparison, it should be noted that zigzag-edge WS_2_ and MoS_2_ NRs are metallic and ferromagnetic^11^,^25^.

**Figure 3:**
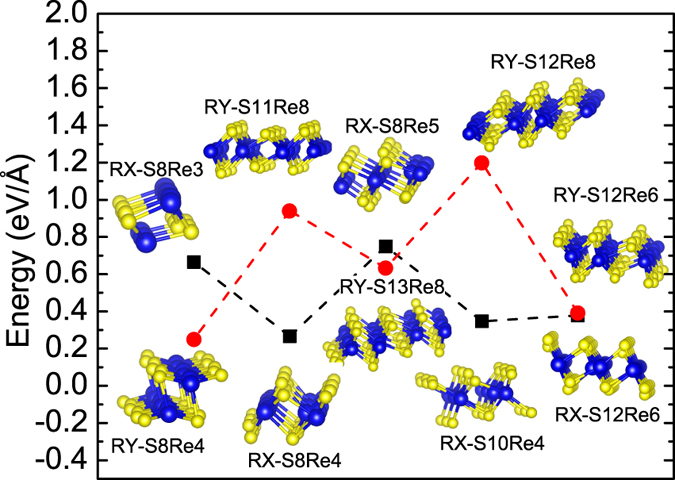
The calculated edge energies of different types of ReS_2_ NRs.

**Figure 4:**
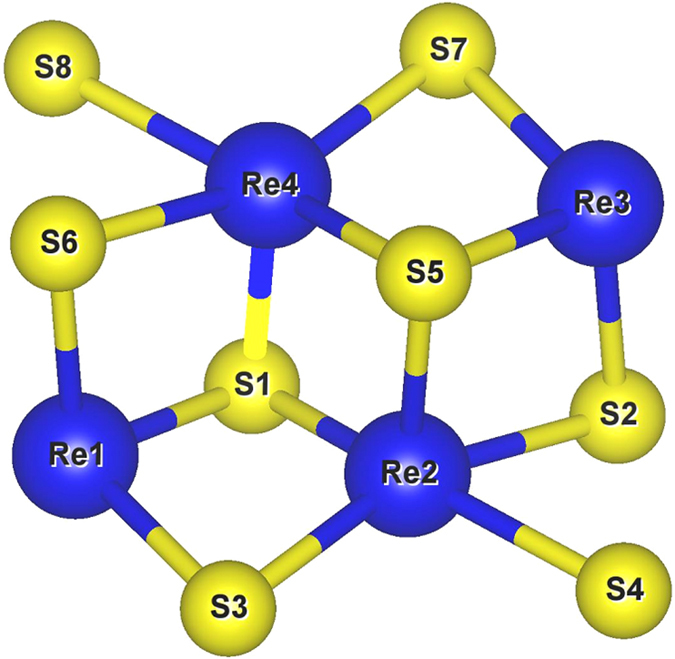
The optimized model of RY-S8Re4 NR unitcell.

The edge energy (*E*_edge_) is an indication of the NR’s stability, and can be calculated based on:


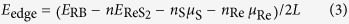


where *E*_RB_ is the total energy of the ReS_2_ NR, and 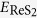 is the energy of ReS_2_ unitcell calculated from the monolayer. *L* is the length of NRs. *n*_S,(Re)_ is the number of extra S or Re atoms at the edges, respectively. *μ*_S,(Re)_ is the chemical potentials of S or Re, respectively. It should be noted that the chemical potentials of Re and S are decided by the thermodynamics condition. For ReS_2_, at equilibrium, we have 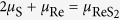, where 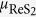 is the chemical potential of ReS_2_^26^. The calculated edge energies of bare NRs as well as 3D view of their corresponding NR configurations are shown in Fig. 3. It is seen that RY-S8Re4 has the minimum edge energy. Consequently, we only focus on exploring the electronic properties of RY-S8Re4 NR. The optimized structure of RY-S8Re4 NR is shown in Fig. 4.

### Size-dependent band structure of nanoribbions

**Figure 5:**
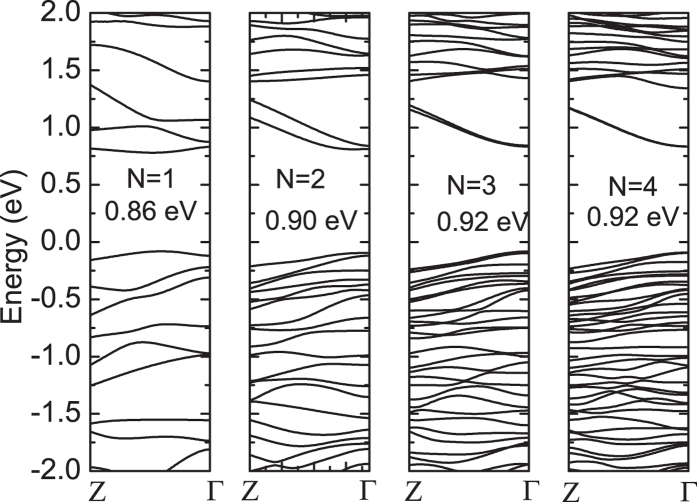
The calculated results of size-dependent band structure of RY1-NRs.

Beyond ML ReS_2_, the electronic properties of ReS_2_ NRs remain largely unexplored, which are crucial for their applications in nanodevices. Here, we focus on the size effect on their electronic properties. It is well-known that NRs (1D) have different electronic properties compared to mono- or multi-layer (2D) form. The edge atoms of NRs introduce new flat energy levels at both valence and conduction band edges, narrowing the band gap accordingly. Here, we further investigate the quantum size effect on the bandgap of ReS_2_ NRs (RY1-series). Our calculation results show that RY1-NRs are semiconductors with a director bandgap, and the bandgap increases with increasing ribbon width (N), and converges to 0.92 eV as shown in Fig. 5. In our models, N = 1, 2, 3, and 4 corresponds to a width of 13.27, 18.27, 20.58, 26.40 and 38.19 Å, respectively. It should be noted that for N = 1, which corresponds to a RY1-NR with a width of 13.27 Å, the *k*-space conduction band minimum and valence band maximum shift away from Γ point. But the NR still retains the nature of direct bandgap, which may be caused by the narrow width and symmetry-breaking in the FBZ.

## Discussion

Figure 2b shows the variation of electron and hole effective masses with biaxial strain in ML ReS_2_ obtained from the DFT calculation results of strain-dependent band structure (see Supplementary Fig. S3 online). The calculated effective masses of electron and hole for strain-free ReS_2_ are 0.83 m_*e*_ and 1.30 m_*e*_, respectively. These values are higher than those of MoS_2_, in which the effective mass for electron is 0.48 m_*e*_ and for hole is 0.60 m_*e*_^10^. From our calculation results as shown in Fig. 2b, we find that electron effective mass is nearly strain-independent, with a value at about 0.83 m_*e*_, corresponding to the strain-free state. Figure 2c shows the variation of total energy with biaxial strain. The in-plane stiffness *C* is calculated using Eq. 2 by fitting the energy–strain curve as shown in Fig. 2c and the value *C* is found to be 353.63 N/m. Figure 2d shows the shift of band edges as a function of biaxial strain. The DP constants *E*_1_ are calculate as *dE*_edge_/*dδ*, equivalent to the slope of the fitting lines as shown in Fig. 2c, where *E*_edge_ is the energy of conduction or valence band edge, and *δ* is the applied strain. The calculated DF constants for electron and hole are −15.17 and −9.5, respectively. We notice that the absolute values of DP constants in ReS_2_ are higher than those in MoS_2_ (~−11 for electron and ~−5 eV for hole^10^, respectively). According to the definition of DP, a higher absolute value of DP constant indicates a more strain-dependent band edge shift. Hence at the same strain level, the band edge shift of ReS_2_ is larger than that of MoS_2_. It should be noted that the band edge shift is not equivalent to the bandgap change. Our calculations show that the bandgap is 1.51 eV at −2% compressive strain and reduces to 1.35 eV at 2% tensile strain. Thus the bandgap difference is only 0.16 eV from −2% compressive to 2% tensile strain (see Supplementary Fig. S4 online). Since the bandgap of monolayer ReS_2_ is insensitive to strain, therefore, ReS_2_ is suitable for flexible electronic device applications.

Based on the obtained values of *E*_1_, *C*, and m^*^, and also Eq. 2, the acoustic phonon-limited charge carrier mobilities of electron and hole at room temperature (T = 300 K) are calculated and listed in Table 1. It is seen that the calculated electron and hole mobilities are 34.21 and 31.09 cm^2^/Vs, respectively. Hence the electron and hole mobilities are very close to each other in ML ReS_2_. Currently, there are no experimental results on the charge carrier mobilities of ML ReS_2_. However, the experimental result of electron mobility in bulk ReS_2_ at room temperature was reported to be 19 cm^2^/Vs^27^. In general, a ML TMD has a lower electron mobility than its bulk counterpart. For example, the bulk MoS_2_ mobility is as high as 200–500 cm^2^/Vs^28^, but the mobility of ML MoS_2_ is only in the range of 0.1–10 cm^2^/Vs^29^,^30^. The low mobility of ML MoS_2_ may potentially limit its applications in practical devices. Although the underlying reason for the reduced mobility of ML MoS_2_ was explained by using the Coulomb scattering model^31^, other mechanisms, such as quantum confinement, defect scattering and substrate effect, may also play an important role in mobility reduction. Currently, how to increase the charge carrier mobility of ML MoS_2_ is still a challenge.

In our study, both bulk and monolayer ReS_2_ forms possess a direct bandgap characteristic and the calculated bandgap of 1.30 eV for bulk ReS_2_ is only slightly lower than that of 1.43 eV for monolayer ReS_2_ as shown in Fig. 2a. The unchanged bandgap characteristic and the small difference in bandgap energy between bulk and monolayer forms clearly indicate that the van der Waals interlayer interaction is weak. Hence the band structure of ReS_2_ is nearly independent of the number of layers. From our calculation results as shown in Fig. 2, it is seen that ML ReS_2_ has nearly the same mobility as its bulk counterpart. This nearly identical mobility in ML and bulk ReS_2_ may be due to the weak interlayer coupling in ReS_2_^12^. Hence, it is expected that ReS_2_ may be more advantageous than other TMDs for making 2D materials-based nanodevices. It is worth noting that our theoretical calculations for mobility are based on perfect ML ReS_2_ without considering any intrinsic or extrinsic defects. In practical fabrication process, native defects such as S vacancies may exist in samples, which can increase free carrier concentration as well as elastic scattering. Hence, the real samples may have a lower mobility than the theoretically predicted value. However, the mobility of ML may be recovered through using high-*k* dielectric. For example, recently, the mobility ML MoS_2_ covered by a high-*k* dielectric layer and metal topgate was reported as high as 900 cm^2^/Vs for ML MoS_2_^31^.

The optimized structure of RY-S8Re4 NR is shown in Fig. 4, in which we find that the zigzag edge of RY-S8Re4 is terminated by S-Re-S atoms (Re-centered-S). The edge termination of the most stable ReS_2_ NR is different compared to other TMD-based NRs, such as MoS_2_ NRs, in which S-terminated zigzag NRs are the most stable without hydrogen saturation^11^. The optimized bond lengths between two nearest atoms in RY-S8Re4 NR are shown in Table 2. For comparison, the optimized bond lengths between two nearest atoms in ML ReS_2_ are shown in brackets. Interestingly, we find that the bond lengths between two nearest atoms in RY-S8Re4 NR are slightly longer than those in ML ReS_2_. No significant distortion is found in the structure optimization of RY-S8Re4 NR.

**Figure 6:**
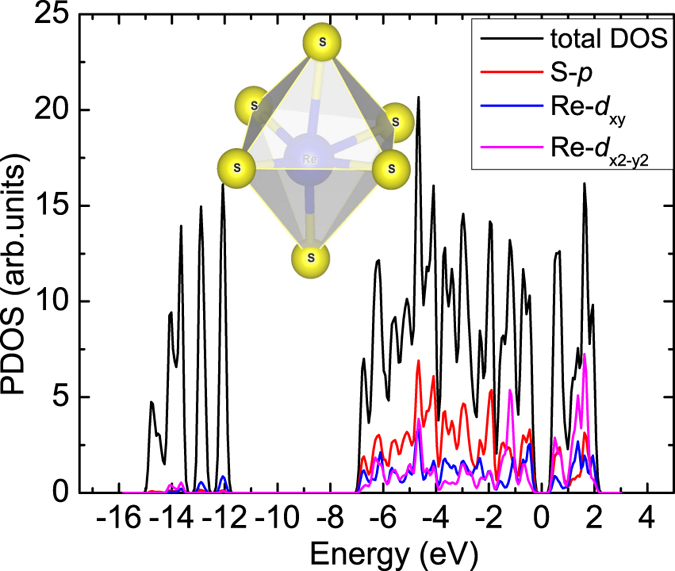
The calculated projected density of states of RY-S8Re4 NR. The inset is the ReS_6_ octahedral complex.

The weak distortion and high stability of RY-S8Re4 NR are due to its unique structure: ReS_2_ has the distorted CdCl_2_-type layer structure, with each Re atom bonding with 6S atoms, forming an octahedral complex (ReS_6_) as shown in the inset of Fig. 6. The slight increase in bond length along the six directions and odd valence electrons from Re (5*d*^5^6*s*^2^) may result in the elongated Jahn-Teller distortions when degeneracy is broken. The *d*^7^ electronic configuration of Re provides one electron in the two degenerate *e*_*g*_ orbitals (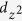 and 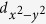), which involve in the degeneracy point directly at S, leading to doubly degenerate electronic ground states. Hence, a distortion may further enhance an energetic stability, which can be proved by analyzing the projected density of states (PDOS) in RY-S8Re4 NR as shown in Fig. 6. It is seen that Re-*d*_*xy*_ and 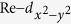 orbitals have significant overlap with S-*p* orbitals. When such an elongation occurs in ReS_2_ NRs, this may push the antibonding orbital Re-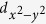 to a higher energy level and bonding orbitals Re-*d*_*xz*_ and/or Re-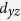 to a lower energy level, causing ReS_2_ NRs to be more energetically stable. In contrast to other TMDs-based NRs, such as MoS_2_ NRs, it was reported that a large distortion was found in edges during the structure optimization even using hydrogen saturation^11^.

Quantum size effects on band structure of ReS_2_ NRs are shown in Fig. 5. Interestingly, the direct bandgap of ReS_2_ NRs finally converges to 0.92 eV in contrast to ML ReS_2_ bandgap of (1.43 eV), which shows that the bandgap of ReS_2_ NR is less strongly dependent on the width than that of other layered TMDs. Compared with other TMD-based NRs, such as MoS_2_ NRs, the bandgap finally converges to 0.56 eV^16^, which is much smaller than their monolayer counterpart (1.90 eV)^32^. Besides, the bandgap of MoSe_2_ NRs with armchair edges reduces to 0.38 eV from their monolayer value 1.55 eV^33^. In some extreme cases, TMD NRs may even show metallic behavior^11^,^16^. Clearly, for ReS_2_ NRs, the reduction in the bandgap compared to the monolayer value is much smaller than other TMDs NRs. It is known that the reduced bandgap may limit the applications of TMDs in electronic nanodevices, especially in optoelectronics, in which the nature and the magnitude of bandgap play critical roles in devices. Hence, we consider that ReS_2_ NRs have more stable structure and more robust direct bandgap characteristic, thus ideal for applications in optoelectronic nanodevices.

In conclusion, we have studied the electronic properties of ML, multilayer and NR forms of ReS_2_. In contrast to other TMDs, ML ReS_2_ has similar behavior as its bulk due to interlayer electronic and vibrational decoupling. We also calculate the mobilities of electron and hole in ML ReS_2_ and find that the mobilities of ML and bulk ReS_2_ are comparable, which is in strong contrast to other TMDs in which a drastic reduction in mobilities from bulk to ML form is often observed. Moreover, we also investigate the quantum size effect on the bandgap of ReS_2_ NRs and find the bandgap is weakly dependent on the NR width, and converges to 0.92 eV for wide NRs. Our work suggests that ReS_2_, with robust sizable and direct bandgap semiconducting characteristics, is a promising material suitable for TMD-based optoelectronic nanodevices.

## Methods

All calculations were carried out using the density functional theory (DFT) with the generalized gradient approximation (PBE-GGA)^34^ and the projector augmented-wave (PAW) pseudopotential plane-wave method^35^, as implemented in the VASP code^36^. For the PAW pseudopotential, we included 5*d*^5^ and 6*s*^2^ valence for Re; for S, the *n* = 3 shell is included as valence (3*s*^2^ and 3*p*^4^). A 10 × 10 × 1 Monkhorst-Pack *k*-point grid was used for monolayered unitcell geometry optimization calculations and a plane-wave basis set with an energy cutoff of 500 eV. A 8 × 1 × 1 grid for *k*-point sampling in geometry optimization was consistently used for NRs in our calculations. Good convergence was obtained with these parameters and the total energy was converged to 1.0 × 10^−5^ eV per atom, as well as edge energy was converged to 1.0 × 10^−3^ eV/Å. For the interlayer coupling energy calculation, we carried out comparative calculations both with and without the van deer Waals correction by employing optB88-vdW functional^37^. In order to discuss the spin polarization effect on the electrical properties of NRs, calculations both with and without spin polarization were carried out to investigate the magnetic properties of the nanoribbons.

## Additional Information

**How to cite this article**: Yu, Z. G. *et al.* Robust Direct Bandgap Characteristics of One- and Two-Dimensional ReS_2_. *Sci. Rep.* **5**, 13783; doi: 10.1038/srep13783 (2015).

## Supplementary Material

Supplementary Information

## Figures and Tables

**Table 1:** Deformation potential E_1_, in-plane stiffness *C*, effective mass m^*^, and mobility *μ* for electron (e) and hole (h) in ML ReS_2_ at 300 K.

**carrier type**	**E**_**1**_**(eV)**	***C*****(N/m)**	m*(m_***e***_)	***μ*** **(cm**^**2**^**/Vs)**
e	15.17	353.63	0.8	34.21
h	9.5	353.63	1.34	33.04

**Table 2:** The optimized bonding lengths in Å between two nearest atoms in RY-S8Re4 NR as shown in Fig. 4.

	**S1**	**S2**	**S3**	**S4**	**S5**	**S6**	**S7**	**S8**
Re1	2.38(2.37)	NA	2.42(2.39)	NA	NA	2.36(2.33)	NA	NA
Re2	2.39(2.37)	2.45(2.43)	2.41(2.39)	2.49(2.45)	2.38(2.36)	NA	NA	NA
Re3	NA	2.36(2.33)	NA	NA	2.38(2.36)	NA	2.42(2.39)	NA
Re4	2.45(2.43)	NA	NA	NA	2.39(2.37)	2.45(2.43)	2.41(2.39)	2.49(2.45)

For comparison, the corresponding bond lengths in ML ReS_2_ are shown in brackets.

## References

[b1] Butler S. Z. *et al.* Progress, Challenges, and Opportunities in Two-Dimensional Materials Beyond Graphene. ACS Nano 7, 2898–2926 (2013). 23464873 10.1021/nn400280c

[b2] Lopez-Sanchez O., Lembke D., Kayci M., Radenovic A. & Kis A. Ultrasensitive photodetectors based on monolayer MoS_2_. Nat. Nanotechnol. 8, 497–501 (2013). 23748194 10.1038/nnano.2013.100

[b3] Baugher B. W. H., Churchill H. O. H., Yang Y. & Jarillo-Herrero P. Optoelectronic devices based on electrically tunable p-n diodes in a monolayer dichalcogenide. Nat. Nanotechnol. 9, 262–267 (2014). 24608231 10.1038/nnano.2014.25

[b4] Pospischil A., Furchi M. M. & Mueller T. Solar-energy conversion and light emission in an atomic monolayer p-n diode. Nat. Nanotechnol. 9, 257–261 (2014). 24608229 10.1038/nnano.2014.14

[b5] Ross J. S. *et al.* Electrically tunable excitonic light-emitting diodes based on monolayer WSe_2_ p-n junctions. Nat. Nanotechnol. 9, 268–272 (2014). 24608230 10.1038/nnano.2014.26

[b6] Yun W. S., Han S. W., Hong S. C., Kim I. G. & Lee J. D. Thickness and strain effects on electronic structures of transition metal dichalcogenides: 2H-MX_2_ semiconductors (M = Mo, W, X = S, Se, Te). Phys. Rev. B 85, 033305 (2012).

[b7] Geim A. K. & Grigorieva I. V. Van der Waals heterostructures. Nature 499, 419–425 (2013). 23887427 10.1038/nature12385

[b8] Zhang H., Li X. & Liu L. Tunable electronic and magnetic properties of WS_2_ nanoribbons. J. App. Phys. 114, 093710 (2013).

[b9] Tongay S., Varnoosfaderani S. S., Appleton B. R., Wu J. & Hebard A. F. Magnetic properties of MoS_2_: Existence of ferromagnetism, Appl. Phys. Lett. 101, 123105 (2012).

[b10] Cai Y., Zhang G. & Zhang Y.-W. Polarity-reversed robust carrier mobility in monolayer MoS_2_ nanoribbons. J. Am. Chem. Soc. 136, 6269–6275 (2014). 24712770 10.1021/ja4109787

[b11] Pan H. & Zhang Y.-W. Edge-dependent structural, electronic and magnetic properties of MoS_2_ nanoribbons. J. Mater. Chem. 22, 7280 (2012).

[b12] Tongay S. *et al.* Monolayer behaviour in bulk MoS_2_ due to electronic and vibrational decoupling. Nature commun. 5, 3252–3257 (2014). 24500082 10.1038/ncomms4252

[b13] Fujita T. *et al.* Chemically exfoliated MoS_2_ nanosheets. Nanoscale 6, 12458–12462 (2014). 25237929 10.1039/c4nr03740e

[b14] Ataca C., Sahin H., Aktürk E. & Ciraci S. Mechanical and electronic properties of MoS_2_ nanoribbons and their defects. J. Phys. Chem. C 115, 3934–3941 (2011).

[b15] Botello-Méndez A. R., López-Urías F., Terrones M. & Terrones H. Metallic and ferromagnetic edges in molybdenum disulfide nanoribbons. Nanotechnology 20, 325703 (2009). 19620764 10.1088/0957-4484/20/32/325703

[b16] Li Y., Zhou Z., Zhang S. B. & Chen Z. MoS_2_ Nanoribbons: High stability and unusual electronic and magnetic properties. J. Am. Chem. Soc. 130, 16739–16744 (2008). 19554733 10.1021/ja805545x

[b17] Ho C. H., Huang Y. S., Liao P. C. & Tiong K. K. Crystal structure and band-edge transitions of ReS_2−*x*_Se_*x*_ layered compounds. J. Phys. Chem. Solids 60, 1797–1804 (1999).

[b18] Marzik J. V., Kershaw R. & Dwight K. Photoelectronic properties of MoS_2_ and MoSe_2_ single crystals. J. Solid State Chem. 51, 170–175 (1984).

[b19] Björkman T., Gulans A., Krasheninnikov A. V. & Nieminen R. M. van der Waals bonding in layered compounds from advanced density-functional first-principles calculations. Phys. Rev. Lett. 108, 235502 (2012). 23003970 10.1103/PhysRevLett.108.235502

[b20] Kaasbjerg K., Thygesen K. S. & Jacobsen K. W. Phonon-limited mobility in n-type single-layer MoS_2_ from first principles. Phys. Rev. B 85, 115317 (2012).

[b21] Kaasbjerg K., Thygesen K. S. & Jauho A.-P. Acoustic phonon limited mobility in two-dimensional semiconductors: Deformation potential and piezoelectric scattering in monolayer MoS_2_ from first principles. Phys. Rev. B 87, 235312 (2013).

[b22] Bardeen J. & Shockley W. Deformation potentials and mobilities in non-polar crystals. Phys. Rev. 80, 72–80 (1950).

[b23] Price P. Two-dimensional electron transport in semiconductor layers. I. Phonon scattering. J. Ann. Phys. 133, 217–239 (1981).

[b24] Xi J., Long M., Tang L., Wang D. & Shuai Z. First-principles prediction of charge mobility in carbon and organic nanomaterials. Nanoscale 4, 4348–4369 (2012). 22695470 10.1039/c2nr30585b

[b25] Ouyang F. *et al.* Effects of edge hydrogenation on structural stability, electronic, and magnetic properties of WS_2_ nanoribbons. J. Appl. Phys. 114, 213701 (2013).

[b26] Schweiger H., Raybaud P. Kresse G. & Toulhoat H. Shape and edge sites modifications of MoS_2_ catalytic nanoparticles induced by working conditions: A theoretical study. J. Catal. 207, 76–87 (2002).

[b27] Tiong K. K., Ho C. H. & Huang Y. S. The electrical transport properties of MoS_2_ and MoSe_2_ layered crystals. Solid State commun. 111, 635–640 (1999).

[b28] Fivaz R. & Mooser E. Mobility of charge carriers in semiconducting layer structures. Phys. Rev. B 163, 743 (1967).

[b29] Novoselov K. S. *et al.* Two-dimensional atomic crystals. Proc. Natl. Acad. Sci. USA 102, 10451–10453 (2005). 16027370 10.1073/pnas.0502848102 PMC1180777

[b30] Ayari A., Cobas E., Ogundadegbe O. & Fuhrer M. S. Realization and electrical characterization of ultrathin crystals of layered transition-metal dichalcogenides. J. Appl Phys. 101, 014507 (2007).

[b31] Radisavljevic B., Whitwick M. B. & Kis A. Integrated circuits and logic operations based on single-layer MoS_2_. ACS Nano 5, 9934–9938 (2011). 22073905 10.1021/nn203715c

[b32] Mak K. F., Lee C., Hone J., Shan J. & Heinz T. F. Atomically thin MoS_2_: A new direct-gap semiconductor. Phys. Rev. Lett. 105, 136805 (2010). 21230799 10.1103/PhysRevLett.105.136805

[b33] Tongay S. *et al.* Thermally driven crossover from indirect toward direct bandgap in 2D semiconductors: MoSe_2_ versus MoS_2_. Nano Lett. 12, 5576–5580 (2012). 23098085 10.1021/nl302584w

[b34] Perdew J. P., Burke K. & Ernzerhof M. Generalized gradient approximation made simple. Phys. Rev. lett. 77, 3865 (1996). 10062328 10.1103/PhysRevLett.77.3865

[b35] Blöchl P. E. Projector augmented-wave method. Phys. Rev. B 50, 17953 (1994). 10.1103/physrevb.50.17953 9976227

[b36] Kresse G. & Furthmüller J. Efficiency of ab-initio total energy calculations for metals and semiconductors using a plane-wave basis set. J. Comput. Mater. Sci. 6, 15 (1996). 10.1103/physrevb.54.11169 9984901

[b37] Klimeš J., Bowler D. R. & Michealides A. Van der Waals density functionals applied to solids. Phys. Rev. B 83, 195131 (2011).

