# Supplementary material for: Robust Direct Bandgap Characteristics of One- and Two-Dimensional ReS2
Source: Sci Rep. 2015 Sep 8;5:13783. doi: 10.1038/srep13783 (PMC4562306; doi:10.1038/srep13783)
Supplement: Supplementary Information [file srep13783-s1.pdf]

Robust Direct Bandgap Characteristics of One- and Two-Dimensional ReS_2

Zhi Gen Yu*, **Yongqing Cai & Yong-Wei Zhang***

Institute of High Performance Computing, Singapore 138632, Singapore.

*Correspondence and requests for materials should be addressed to Z. G. Y. (email: yuzg@ihpc.a-star.edu.sg) or Y-W. Z. (email: zhangyw@ihpc.a-star.edu.sg)

Supporting information

1 The computational models of ReS_2 NRs

ReS_2 NRs can be directly produced by cutting ML ReS_2 along x or y direction. Here, we only illustrate how to obtain the RY-series NRs as shown in Fig. S1. For RX-series NRs, just follow the similar method to cut ML ReS_2 along x direction.

2 Density of states (DOS) of ReS_2 NR with spin polarization

We calculate density of states of all possible NRs with spin polarization, and we find that calculated magnetic moments are zero and the ground-state energies with and without spin polarization are identical. For illustration, we only plot the DOS of RY-S8Re4 NR as shown in Fig. S2.

3 Band structures of ML ReS_2 under varied biaxial strains

We calculate the band structure of ML ReS_2 as a function of biaxial strains in the range of -2% compressive strain to 2% tensile strain, and the band structure variation under strain is addressed in Fig. S3. It is seen that the compressive strain of -2% leads to a direct-to-indirect bandgap transition. Which may also cause the unexpected increase in the hole effective mass. Generally, the band structures of 2D materials are sensitive to the compressive strain and insensitive to the tensile strain. Such as, for WS_2, only -1% compressive strain leads to a direct-to-indirect bandgap transition [1]. For tensile strain effect on the nature of bandgap, it is seen that the nature of the bandgap remains direct. We also extend the range of the tensile strain to 5% in order to find the critical value of the nature of bandgap transition. When the tensile strain increases to 4.5%, a transition from a direct-to-indirect bandgap is achieved for ReS_2. In contrast to other TMDs, the transition from a direct-to-indirect bandgap is achieved for the tensile strain of 1% for MoS_2 and 1.8% for WS_2 [2,3]. Therefore, ReS_2 is a promising material suitable for TMD-based optoelectronic nanodevices.

4 Summarized Band gaps of ML ReS_2 as a function of biaxial strains

The summarized bandgaps of ML ReS_2 as a function of biaxial strains are shown in Fig. S4 according to the calculated band structures as shown in Fig. S3.

References

1. Amin B., Kaloni T. P. & Schwingenschlögl U. Strain engineering of WS_2, WSe_2, and WTe_2. *RSC Adv.* **4**, 34561, (2014).
2. Conley H.J. *et al*. Bandgap Engineering of Strained Monolayer and Bilayer MoS_2. *Nano Lett.* **13**, 3626-3630, (2013).
3. Ghorbani-Asl M., Borini S., Kuc A. & Heine T. Strain-dependent modulation of conductivity in single-layer transition-metal dichalcogenides. *Phys. Rev. B* **87**, , 235434, (2013).

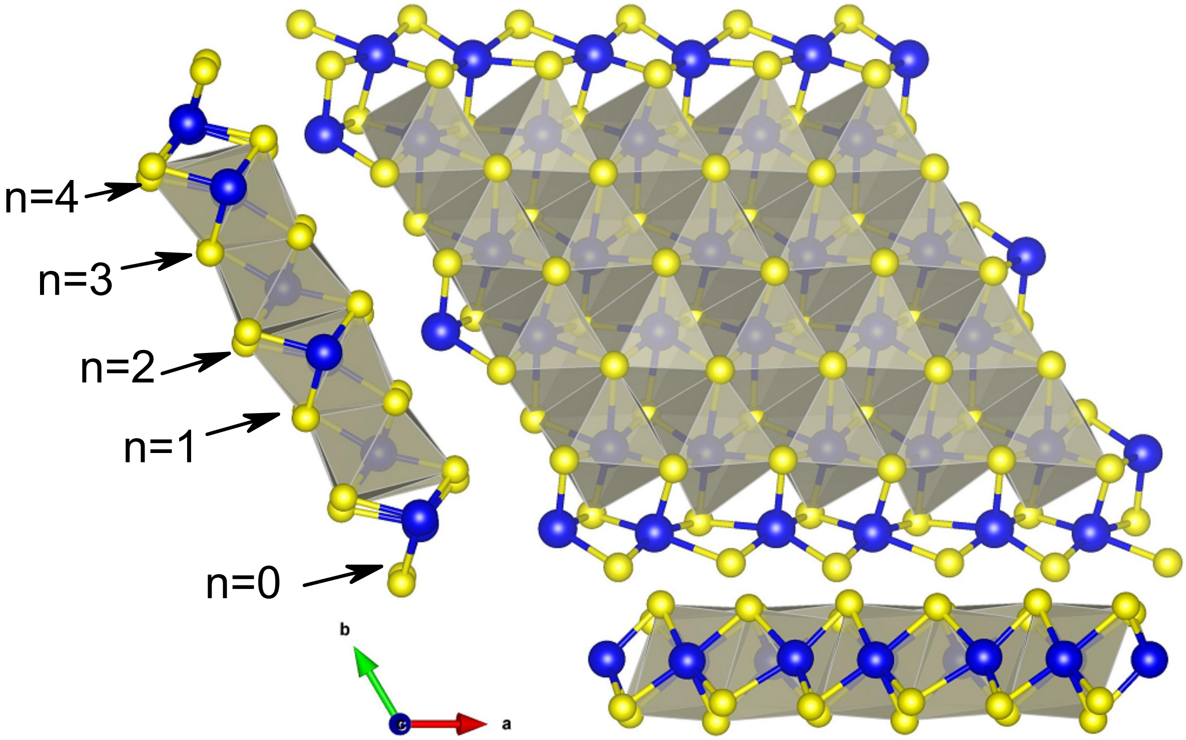

Figure S1. The models of ML ReS_2 (top right) and RY-series NRs (top left). The bottom right model shows the side view of ML ReS_2.

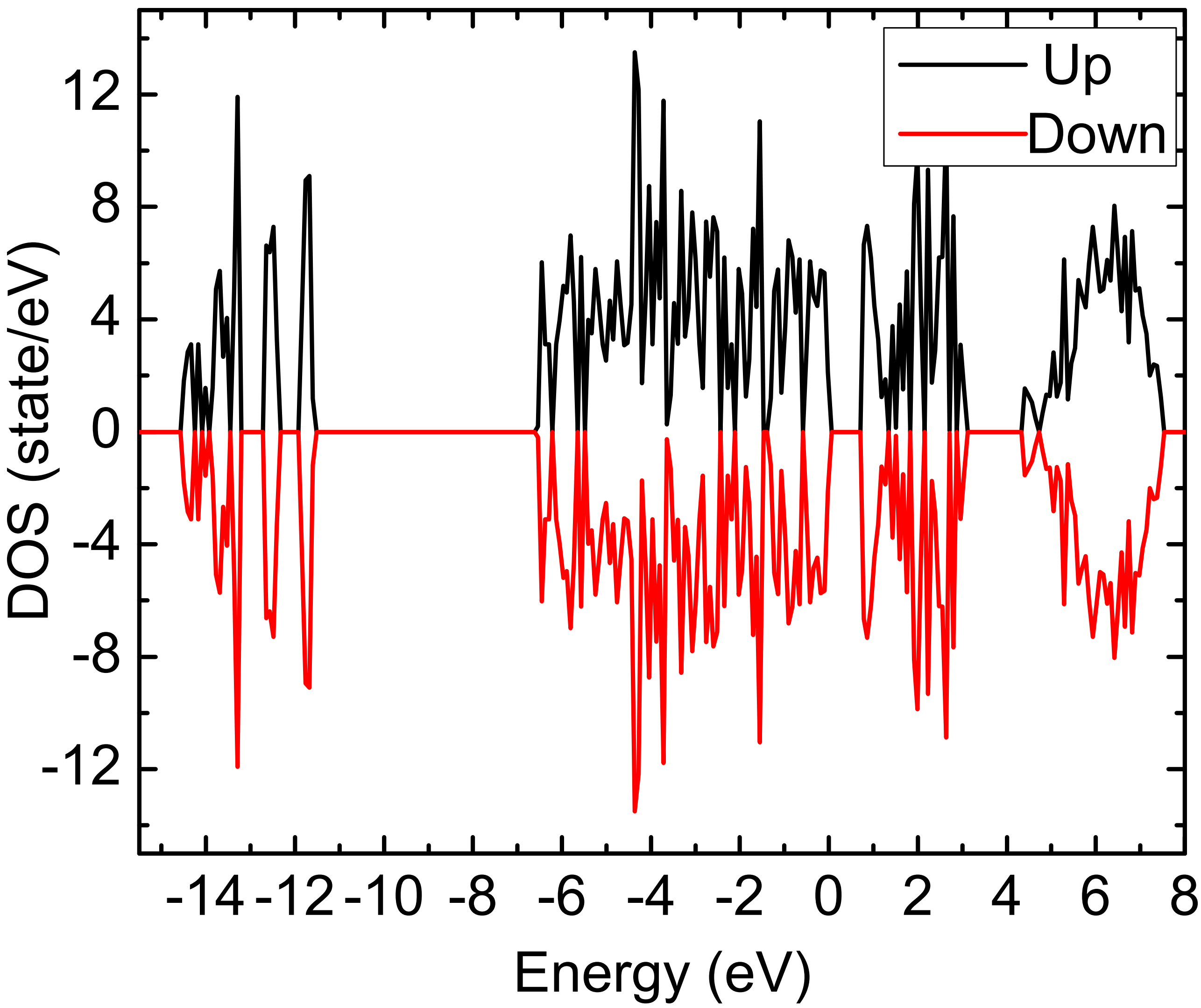

Figure S2. Density of states (DOS) of ReS_2 NR (RY-S8Re4) with spin polarization.

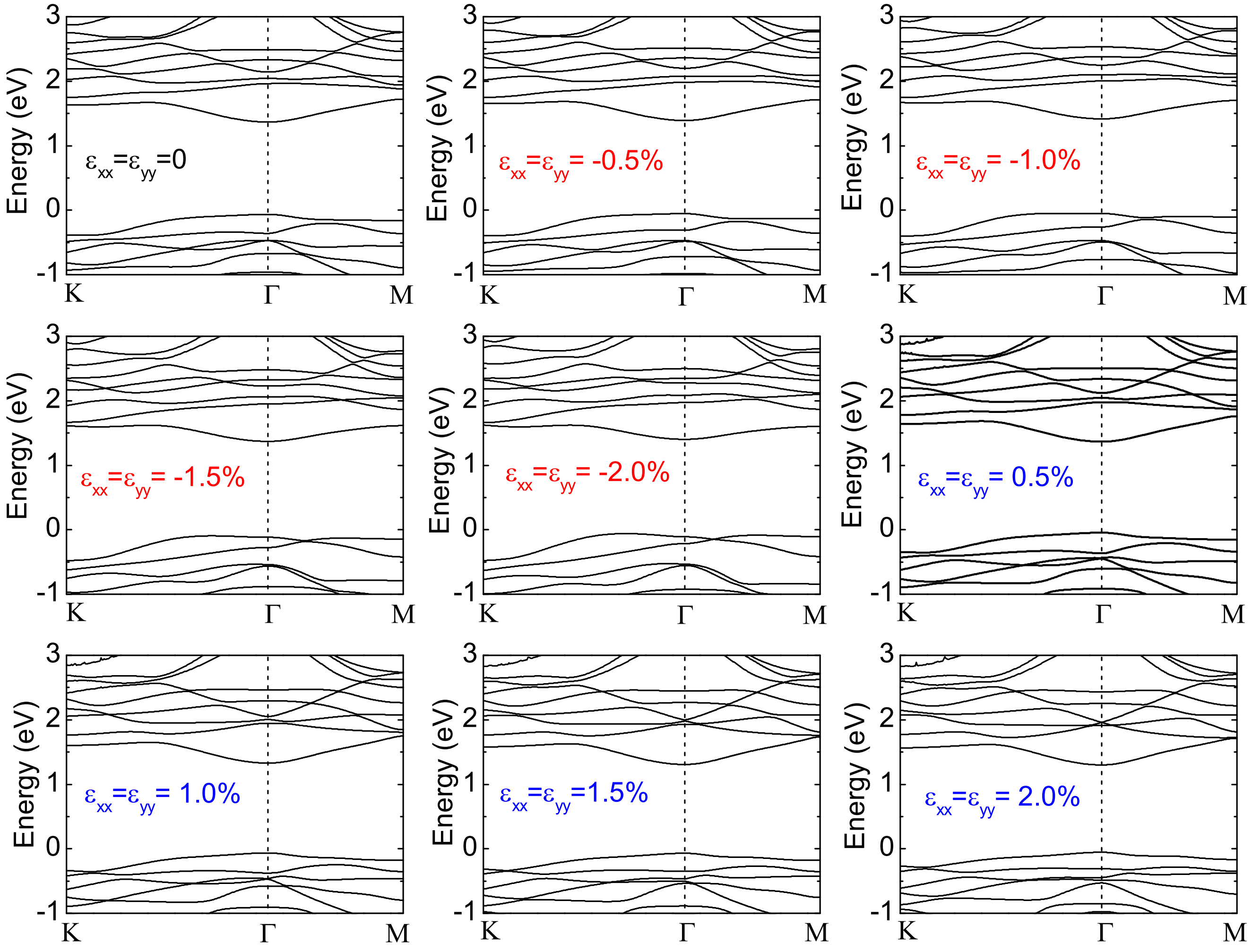

Figure S3. The calculated band structures of ML ReS_2 under varied biaxial strains.

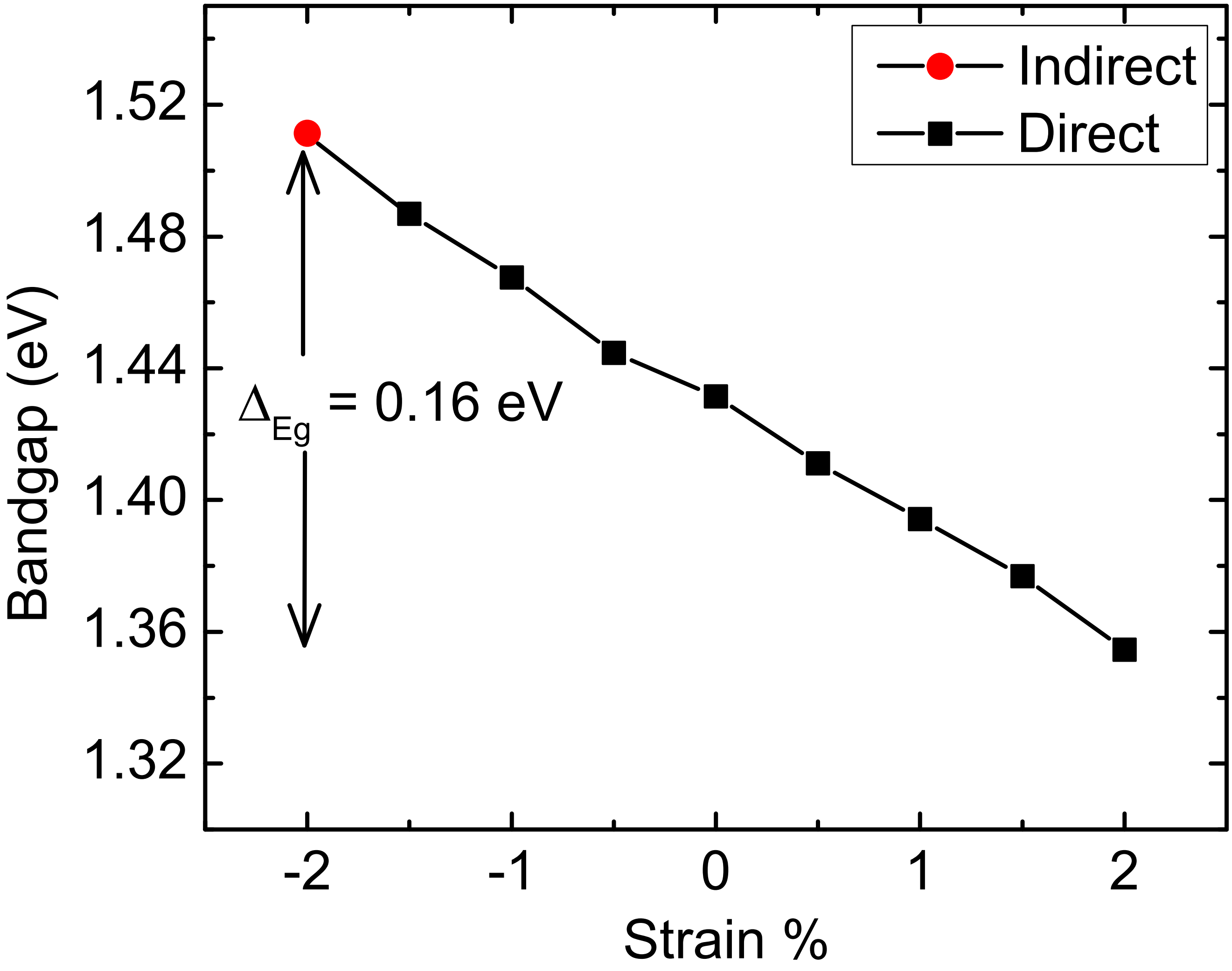

Figure S4. The calculated bandgaps of ML ReS_2 under varied biaxial strains.
